# Hydrophobic Ion Pairing Improves Protein Thermal Stability and Lipid Nanocarrier Integration through Controlled Surface Modification

**DOI:** 10.1021/acsnanoscienceau.6c00033

**Published:** 2026-05-12

**Authors:** Jiaming Mu, Daniel Sedough-Abbasian, Leran Mao, Sheiliza Carmali

**Affiliations:** † School of Pharmacy, 1596 Queen’s University Belfast, Belfast BT9 7BL, United Kingdom; ‡ Department of Chemical Engineering, 6612 Carnegie Mellon University, Pittsburgh, Pennsylvania 15213, United States

**Keywords:** hydrophobic ion-pairing, protein surface engineering, protein−lipid interactions, nanostructured lipid carriers, lipid crystallinity, protein encapsulation, protein thermal stability

## Abstract

Integrating therapeutic proteins into lipid-based nanocarriers remains challenging due to fundamental incompatibilities between hydrophilic protein surfaces and lipophilic carrier matrices. Here, we demonstrate that controlled hydrophobic ion pairing (HIP) using lysozyme-sodium dodecyl sulfate (SDS) at stoichiometric ratios simultaneously improves thermal stability during pharmaceutical processing and lipid compatibility for nanocarrier integration. Building on our previous molecular characterization of lysozyme-SDS complexes, we investigated their application in nanostructured lipid carrier (NLC) formulations for oral protein delivery. SDS complexation preserved lysozyme enzymatic activity at processing-relevant temperatures and increased apparent lipid miscibility by 50% in both solid and liquid pharmaceutical lipid excipients. This enhanced compatibility translated directly into superior NLC performance, with complexed lysozyme achieving a 4-fold higher encapsulation efficiency (79.6 ± 1.8% vs 18.4 ± 4.3% for native protein) with improved batch-to-batch reproducibility. In simulated gastrointestinal conditions, lysozyme-SDS NLCs demonstrated sustained intestinal release (87.2 ± 16.5% over 6 h) with 67.5 ± 7.5% enzymatic activity retained compared to only 15.2 ± 3.0% activity for native lysozyme formulations. Mechanistic analysis from differential scanning calorimetry and crystallinity measurements showed that SDS complexation induces a molten globule-like protein state that reduces lipid packing (56.8% vs 68.6% crystallinity for native lysozyme), enabling superior matrix integration while maintaining biological function. These findings establish that controlled surface modification through HIP provides a systematic approach to overcome protein–lipid incompatibilities, offering a generalizable framework for developing lipid-based protein therapeutics.

## Graphical abstract

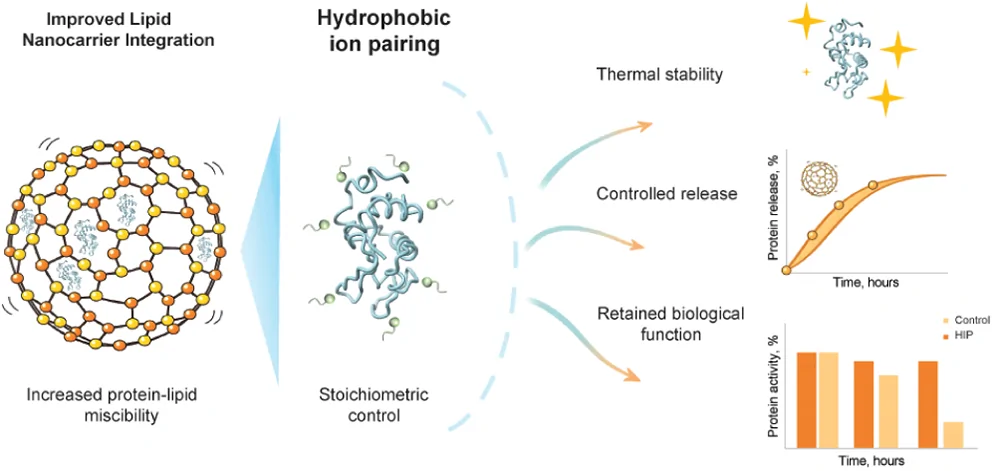

## Introduction

Protein–lipid interactions govern numerous biological processes and have profound implications for therapeutic delivery systems.^1–3^ In nature, these interactions occur through electrostatic forces, hydrogen bonding, and hydrophobic effects, creating dynamic interfaces that maintain both structural integrity and functional flexibility.^4^ Harnessing these principles for pharmaceutical applications remains challenging, particularly when engineering proteins with enhanced stability while preserving their function.

Over the last few decades, sophisticated tools for modifying protein surface properties have been developed, opening new avenues for probing and engineering biological systems.^5,6^ However, traditional bioconjugation strategies rely on covalent modifications that can compromise the protein structure and function. Noncovalent approaches, such as hydrophobic ion pairing (HIP), offer attractive alternatives by providing reversible modifications that impart non-native physicochemical characteristics.^7,8^

HIP involves the stoichiometric association between ionizable groups on proteins and oppositely charged surfactants, creating amphiphilic complexes with altered interfacial behaviors.^9,10^ While this technique shows promise for modulating protein lipophilicity, its effects on protein stability, activity, and interactions with complex lipid environments remain poorly understood at the molecular level.^10,11^ Elucidating these structure–function relationships is essential for establishing its broader utility in protein engineering applications.

Nanostructured lipid carriers (NLCs) are sophisticated lipid-based delivery systems that could benefit significantly from enhanced protein–lipid compatibility.^12^ These carriers, composed of mixtures of solid and liquid lipids, create matrices with controlled imperfections that accommodate therapeutic molecules while providing protection and controlled release properties.^13^ However, the inherent hydrophilicity of most proteins and peptides creates fundamental incompatibilities with lipid environments, requiring advanced manufacturing approaches or chemical modification strategies.^14,15^ Moreover, NLC preparation typically requires elevated temperatures for lipid melting and homogenization, necessitating protein stability under processing conditions.

Recent advances in understanding protein–surfactant interactions have shown that controlled surfactant complexation can retain and even enhance protein function when surfactant concentrations and chemistries are carefully optimized.^10,16–18^ Our previous work demonstrated that lysozyme-SDS interactions at substoichiometric ratios improved enzymatic activity through favorable conformational changes, suggesting the formation of functionally superior protein states.^10^ Building on these molecular insights, we here investigated whether these modifications translate into enhanced protein–lipid compatibility and improved integration within nanocarrier systems, thereby addressing a key requirement for robust protein delivery platforms. In the context of oral administration, protein formulations face the hostile gastrointestinal environment, where acidic pH and proteolytic enzymes rapidly destabilize unprotected proteins. Lipid-based carriers must therefore achieve both a high protein encapsulation efficiency and effective protection during gastrointestinal transit. Using lysozyme as a well-characterized model protein, we demonstrate that controlled SDS complexation simultaneously enhances thermal stability, improves lipid compatibility, and markedly increases nanocarrier encapsulation efficiency, while preserving biological activity.

## Experimental Section

### Materials and Methods

Lysozyme from chicken egg white (lyophilized powder, ≥20,000 units/mg) and the Micro BCA Protein Assay Kit were purchased from Thermo Fisher Scientific. Sodium dodecyl sulfate (SDS), dioctyl sulfosuccinate (DOSS), dimethyl sulfoxide (DMSO), sodium acetate, acetic acid (≥99%), potassium phosphate monobasic solution, potassium phosphate dibasic solution, maleic acid (≥99%), and *Micrococcus lysodeikticus* lyophilized cells were obtained from Sigma-Aldrich. Glyceryl dipalmitate/distearate (Precirol ATO 5) and propylene glycol monocaprylate (Capryol 90) were kindly donated by Gattefossé, France. Polysorbate 80 (Tween 80) was kindly donated by CRODA, UK. The HiTrap SP HP cation exchange column (1 mL) was purchased from Cytiva (Uppsala, Sweden). Vivaspin 20 centrifugal concentrators (1000 kDa MWCO, poly­(ether sulfone) membrane) were purchased from Sartorius (Göttingen, Germany). All chemicals were used without further purification. Deionized water (18.2 MΩ·cm, Milli-Q system) was used for all the experiments.

### Lysozyme-Surfactant Complexation

Hydrophobic ion-pairing between lysozyme and surfactants was performed as previously reported.^10^ A lysozyme solution (5 mg/mL) was prepared in 10 mM acetate buffer at pH 4.5 to obtain a net positive charge for optimal complexation. A surfactant aqueous solution (6.3 mM, 1 mL) was added dropwise to 1 mL lysozyme solution and mixed for 20 min at 550 rpm (Eppendorf 5382 ThermoMixer C v.3.5.0) at 25 °C to achieve a 1:1 surfactant:lysozyme charge ratio complexation. The resulting cloudy suspension indicated complex formation. Lysozyme complexes were recovered by centrifugation at 10,000 rpm for 10 min at 4 °C (Axyspin refrigerated microcentrifuge), yielding white precipitates. The precipitates were washed with deionized water to remove unbound surfactant, lyophilized (Edwards Modulyo Freeze-Dryer), and stored at −20 °C.

### Thermal Stability Analysis

Lysozyme complexes were suspended in 50 mM potassium phosphate buffer, pH 6.5, containing 1.0 M NaCl, and allowed to mix at 550 rpm for 6 h for dissociation, followed by centrifugation at 10,000 rpm for 10 min at 4 °C. The supernatant was collected and analyzed using the Micro BCA assay to determine the concentration of noncomplexed lysozyme. Dissociated lysozyme was diluted to 5 μg/mL (0.35 μM) using 50 mM phosphate buffer, pH 6.5. Samples were incubated at 70 °C for 2, 10, 30, and 60 min, with native lysozyme serving as the control. Lysozyme thermal stability was assessed by measuring residual enzymatic activity at each time point using the *M. lysodeikticus* cell wall assay and expressed as units/mL.^19^ Here, one unit of enzyme activity is defined as the amount of enzyme required to decrease absorbance at 450 nm by 0.001 per min at pH 6.5 and 25 °C, using *M. lysodeikticus* suspension as the substrate in 50 mM potassium phosphate buffer. All measurements were performed in triplicate.

### Lipid Solubility Studies

The solubility of lysozyme and lysozyme-SDS complexes in pharmaceutical lipids was evaluated using hot-stage polarized light microscopy.^20,21^ Lysozyme or lysozyme-SDS complex was dissolved in glyceryl dipalmitate/distearate, and propylene glycol monocaprylate at varying concentrations (0.5, 1.0, 2.5, and 5.0 mg/mL) via magnetic stirring at 200 rpm at 70 °C for 15 min. Subsequently, 50 μL of each mixture was placed on 24 × 24 mm coverslip slides, positioned on a hot-stage microscope (Olympus BX50), and heated at 30 °C/min to 85 °C. Images were collected at 10× magnification under polarized light. Saturation solubility was determined as the highest concentration at which no birefringent crystals were observed under polarized light after heating.^22^ All measurements were performed in triplicate.

### NLC Preparation

NLCs were produced using combined high-shear homogenization and probe sonication.^23^ Formulations comprised a 70:30 ratio of solid to liquid lipid, with detailed compositions provided in Table 1.

**Table 1:** Composition of Nanostructured Lipid Carriers (Blank NLCs—Control, L-NLCs—Lysozyme-Loaded NLCs, and L-SDS NLCs—Lysozyme-SDS Complex-Loaded NLCs)^a^

Component	Blank NLCs	L-NLCs	L-SDS NLCs
***Lipid phase***			
Glyceryl distearate/dipalmitate	85 μL	85 μL	85 μL
Propylene glycol monocaprylate	38 μL	38 μL	38 μL
Lysozyme	-	61.5 μg	-
Lysozyme-SDS complex	-	-	123 μg
***Aqueous phase***			
Polysorbate 80	60 mg	60 mg	60 mg
Water	4 mL	4 mL	4 mL

a Protein loading amounts were based on saturation solubilities determined from hot-stage microscopy studies. All formulations were prepared in triplicate.

For blank NLCs, glyceryl dipalmitate/distearate was melted at 70 °C, after which propylene glycol monocaprylate was added and allowed to stir at 200 rpm for 15 min. For protein-loaded NLCs, native lysozyme or lysozyme-SDS complex was first dissolved in propylene glycol monocaprylate at 70 °C for 7.5 min with stirring at 200 rpm, after which glyceryl dipalmitate/distearate was added and mixed for another 7.5 min. Protein loading levels (61.5 μg for native lysozyme, 123 μg for lysozyme-SDS complex) were based on saturation solubilities determined from hot-stage microscopy studies (0.5 and 1.0 mg/mL in the total lipid phase, respectively), ensuring maximum protein incorporation while avoiding supersaturation and precipitation.

Separately, polysorbate 80 was dissolved in deionized water to prepare a 1.5% w/v surfactant solution. Both phases were maintained at 70 °C and homogenized using a PowerGen 500 high-shear homogenizer (Thermo Fisher Scientific) at 12,333 rpm for 4 min, followed by probe sonication (Fisherbrand CL-18) at 40% amplitude with 5-s on/off cycles for 10 min total sonication time. The hot nanoemulsion was rapidly cooled in a 4 °C water bath under magnetic stirring (200 rpm, 10 min) to promote lipid crystallization. Fresh NLC dispersions were prepared in triplicate and characterized within 24 h.

### Differential Scanning Calorimetry (DSC)

Thermal analysis was performed using a DSC Q20 (TA Instruments, USA). Samples (3–5 mg) in sealed aluminum pans were analyzed under a nitrogen atmosphere, equilibrated at −20 °C for 5 min, then heated from 20 to 200 °C followed by a cooling to −20 °C at a constant rate of 10 °C/min. An empty aluminum pan served as reference.

Native lysozyme and lysozyme-SDS complexes were analyzed as lyophilized powders, while lipids were analyzed directly without further treatment. For NLC formulations, dispersions were lyophilized for 48 h (Edwards Modulyo Freeze-Dryer) prior to analysis.

Thermograms were analyzed using OriginPro 2021 (OriginLab Corporation, USA). Melting temperatures (*T*_m_) were determined from endothermic peak maxima, crystallization temperatures (*T*_c_) from exothermic peak maxima, and melting enthalpies (ΔH) from integrated peak areas.

Crystallinity index for NLC formulations was calculated relative to pure glyceryl distearate/dipalmitate (solid lipid, eq 1).

1 $$Crystallinity\;Index,\%=\frac{\Delta H_{sample}}{0.69\times \Delta H_{solid\;lipid}}\times 100$$

Where Δ*H*_*solid lipid*_ = 28.3 ± 1.0 J/g, and 0.69 corresponds to the solid lipid fraction in the formulation. All measurements were performed in triplicate, and results are reported as mean ± standard deviation.

### Particle Size, Dispersity, and Zeta Potential Measurements

Nanoparticle size, polydispersity index (PDI), and zeta potential were measured by dynamic light scattering (DLS) using a Zetasizer Nano ZS (Malvern Instruments Ltd., Malvern, UK). NLC formulations were diluted 4-fold with deionized water and filtered through 0.45 μm hydrophilic polytetrafluoroethylene (PTFE) membrane filters. Measurements were conducted at 20 °C using a refractive index of 1.462. Zeta potential was determined by laser Doppler microelectrophoresis. All analyses were performed in triplicate, and results are reported as mean ± standard deviation.

### Determination of Encapsulation Efficiency and Drug Loading

Encapsulation efficiency (EE) and drug loading (DL) were determined by ultrafiltration-centrifugation followed by quantification of nonencapsulated protein.

Fresh NLC dispersions were centrifuged through 1,000 kDa MWCO centrifugal devices (Vivaspin20, approximate pore size ∼20 nm) at 3,000 *g* and 4 °C for 150 min (Harrier 18/80 refrigerated centrifuge). This MWCO was selected to ensure the free passage of nonencapsulated lysozyme during centrifugation (Supporting Information, Table S8). Free (nonencapsulated) lysozyme in the obtained filtrate was quantified using cationic exchange chromatography (ÄKTA start, HiTrap SP HP column) to avoid interference from lipids and surfactants. Samples were eluted with a 0–1 M NaCl gradient in 20 mM sodium phosphate buffer at pH 6.8, detected at 280 nm, and quantified using a calibration curve (*R* ^2^ = 0.9998, LOD = 7 μg/mL, see Supporting Information Figure S1).

Encapsulation efficiency and drug loading were calculated as

2 $$EE,\;\%=\frac{Lyz_{Total}-Lyz_{Nonencapsulated}}{Lyz_{Total}}\times 100$$

3 $$DL,\;\mu g/mg=\frac{Lyz_{Total}-Lyz_{Nonencapsulated}}{Mass_{Total}}\times 100$$

where *Lyz*_*Total*_ is the initial lysozyme mass (μg), *Lyz*_*Nonencapsulated*_ free lysozyme in filtrate (μg), and *Mass*_*Total*_ refers to the total mass in the formulation (lipids and lysozyme mass combined; Supporting Information, Table S5). Surfactant mass was not included in the calculation as it functions as a stabilizer rather than a lipid core constituent. Measurements were performed using three independent NLC batches.

### NLC In Vitro Release and Stability Study

Lysozyme release from NLCs was evaluated in simulated gastrointestinal fluids to assess stability and release kinetics under physiologically relevant pH conditions. Simulated gastric fluid (SGF) was prepared according to USP guidelines by dissolving 2.0 g NaCl and 7.0 mL concentrated HCl (37%) in deionized water, diluting to 1 L, and adjusting to pH 1.2 ± 0.1.^24^ Simulated intestinal fluid (SIF) consisted of 50 mM potassium phosphate buffer at pH 6.8 ± 0.1.^25^ Pepsin and pancreatin were omitted from SGF and SIF, respectively, to isolate the effects of pH on protein release and stability without enzymatic degradation.

Fresh NLC dispersions were added to the release medium (2.5% w/v total lipid concentration, 42.5 mL, Supporting Information, Table S5) and incubated at 37 °C under gentle shaking (100 rpm, Incu-Shake MINI, SciQuip, ISO9001). At predetermined time points (30, 60, and 120 min for SGF; 30, 60, 180, and 360 min for SIF), 3 mL aliquots were withdrawn and replaced with fresh medium to maintain sink conditions. Sample aliquots were centrifuged through 1,000 kDa MWCO centrifugal devices (Vivaspin20) at 3,000 *g* and 4 °C for 180 min (Harrier 18/80 refrigerated centrifuge) to separate released lysozyme from intact NLCs.^23^ The filtrate (1 mL) was collected and analyzed by cationic exchange chromatography using three independent NLC batches.

Released lysozyme collected from the ÄKTA fraction collector was normalized (L-NLC 1 μg/mL, L-SDS NLC 3 μg/mL) to enable comparison of specific activity between formulations with different release concentrations. Normalized lysozyme (80 μL) was added to *M. lysodeikticus* suspension (800 μL, 0.3 mg/mL) in 50 mM phosphate buffer, pH 6.5, and absorbance at 450 nm was monitored at 15-s intervals at 25 °C using a microplate spectrophotometer (Thermo Scientific Multiskan SkyHigh).^19^ Native lysozyme at equivalent concentrations, freshly prepared in SGF or SIF (*t* = 0), served as controls representing 100% activity. Residual activity at each time point was calculated relative to these *t* = 0 controls as

4 $$Residual\;Activity,\;\%=\frac{Released\;Lyz_{ti}}{Control\;Lyz_{t0}}\times 100$$

### Molecular Dynamics (MD) Simulations and Analysis

Simulations were performed using GROMACS 2020.1,^26^ following previously described protocols with minor adjustments.^10^ Briefly, protein systems were solvated using TIP3P explicit solvent and neutralized with 50 mM Na^+^ and PO_4_^2–^ ions. The lysozyme structure was energy-minimized using the steepest-descent algorithm for 5,000 steps, followed by NVT equilibration for 125 ps with Nosé–Hoover temperature coupling. Production simulations, with and without surfactant, were then run for 50 ns with an NPT ensemble using Nosé–Hoover temperature coupling and Parrinello–Rahman isotropic pressure coupling at 293.15 K and 343.15 K. Trajectories were analyzed for root mean squared deviation (RMSD) and radial probability distribution (*g*(*r*)) using Visual Molecular Dynamics.^27^ The average structure from the final 5 ns of the simulation was exported as a PDB file and visualized in Biovia Discovery Studio (Dassault Systems) for secondary structure analysis.

### Statistical Analysis

Means and standard deviations were calculated with Microsoft Excel (Microsoft, Redmond, WA). Results were statistically analyzed by one-way or two-way analysis of variance (ANOVA) using GraphPad Prism version 10.4.2 for Mac, GraphPad Software (Boston, Massachusetts, USA, www.graphpad.com).

## Results and Discussion

### SDS Complexation Retains Lysozyme Function at Processing Temperatures

Protein thermal stability is an important parameter for both biological function and pharmaceutical applications.^28^ The molecular interaction forces governing thermal stability, such as hydrogen bonding networks, hydrophobic interactions, and electrostatic contributions, can be modulated through controlled chemical modifications.^29,30^ To evaluate how surfactant complexation affects protein thermal resistance, we investigated lysozyme stability before and after complexation with two anionic surfactants, sodium dodecyl sulfate (SDS) and dioctyl sulfosuccinate (DOSS) at 70 °C, a temperature relevant for lipid nanocarrier processing.^31^ Thermal challenge was applied following dissociation to assess whether complexation induced a protective state.

Heat denaturation studies showed distinct thermal stability patterns among native and its surfactant complexes (Figure 1). Both native lysozyme and lysozyme-SDS complexes demonstrated comparable thermal resistance at 70 °C, with activity levels remaining statistically equivalent throughout the 60 min heating period (*p* > 0.05, two-way ANOVA, Figure 1a). In contrast, DOSS complexation resulted in substantially reduced baseline activity (1833 ± 115 units/mL at 2 min, representing 72% lower activity than native lysozyme, *p* < 0.001), with activity remaining impaired throughout the 60 min heating period (1522 ± 138 units/mL at 60 min, *p* < 0.01). Comparative end point analysis at 60 min confirmed these divergent behaviors (Figure 1b). While SDS complexation maintained 85% of native lysozyme activity (*p* > 0.05), the DOSS complex retained only 29% of native activity (*p* < 0.0001).

**Figure 1:**
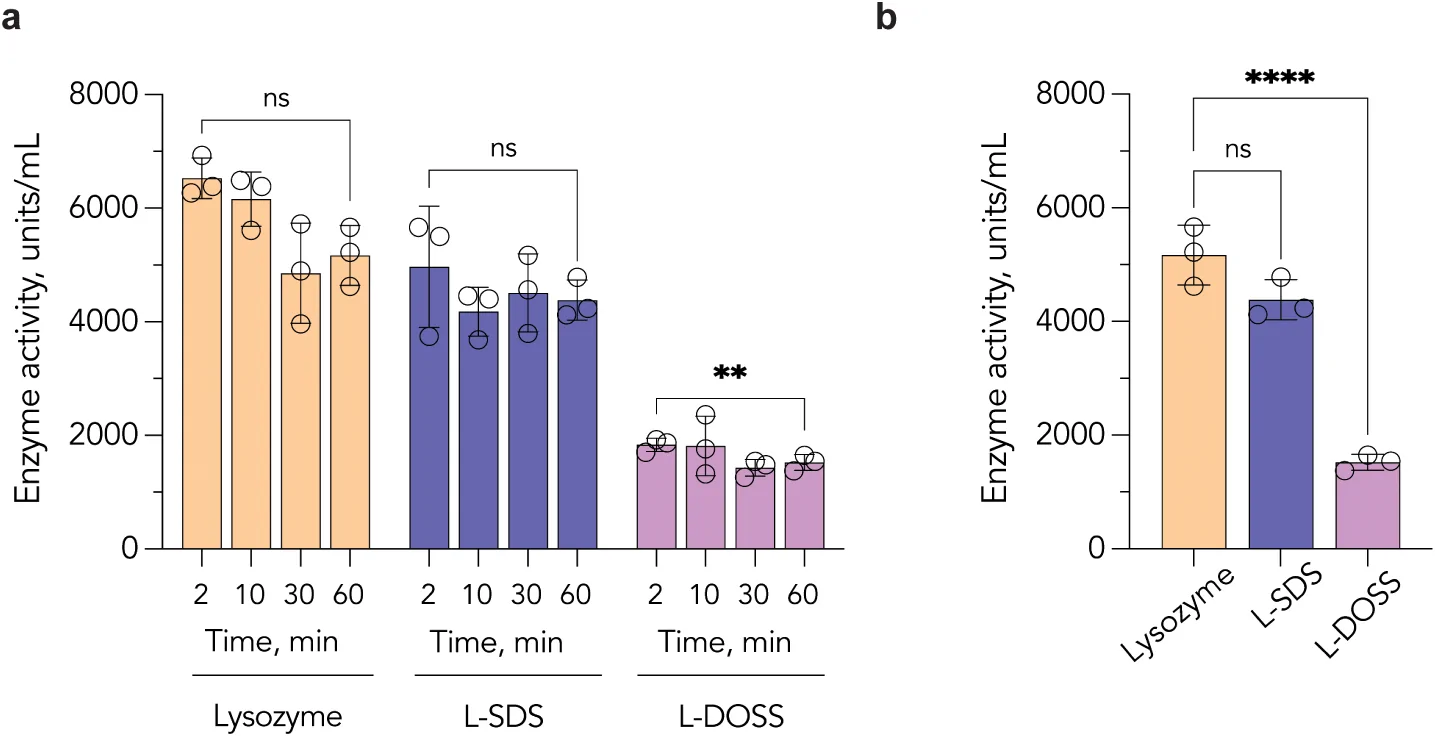
Thermal stability of native lysozyme and lysozyme-surfactant complexes at 70 °C. **a** Enzyme activity of native lysozyme, lysozyme-SDS complex (L-SDS), and lysozyme-DOSS complex (L-DOSS) monitored over 60 min of incubation at 70 °C. Both native and L-SDS complexes retained enzymatic activity throughout the heating period (ns, *p* > 0.05), while L-DOSS complexes showed consistently lower overall activity (**, *p* < 0.01). **b** Comparative analysis of enzymatic activity for native lysozyme, L-SDS, and L-DOSS after 60 min of exposure to 70 °C. L-SDS complexes retained comparable activity to native lysozyme (ns, *p* > 0.05), while L-DOSS complexes showed significantly reduced activity (****, *p* < 0.0001 compared to native lysozyme). Data are presented as mean ± SD from three independent experiments. Statistical significance was determined using two-way ANOVA with Tukey’s post hoc test for panel **a**, and ordinary one-way ANOVA with Dunnett’s multiple comparisons test for panel **b**.

The thermal resistance observed with SDS complexation can be attributed to synergistic molecular mechanisms. Ionic interactions between SDS sulfate groups and lysozyme’s positively charged residues create additional stabilizing networks that reinforce protein structure.^32^ Importantly, complexation at the stoichiometric 1:1 charge ratio employed here induces beneficial conformational reorganization rather than complete denaturation.^10^

Molecular dynamics simulations at 70 °C provided mechanistic insights into the contrasting behaviors of SDS and DOSS complexes (Supporting Information, Figures S2–S4). DOSS complexation resulted in substantially greater structural fluctuation (root-mean-square deviation, RMSD) compared to both SDS complexation and native lysozyme, alongside a loss of α-helical content (Supporting Information, Figures S2 and S3). Radial distribution analysis further showed that DOSS molecules clustered more closely around the catalytic residues Glu35 and Asp52 at 70 °C, potentially occluding the active site (Figure S4). In contrast, SDS maintained a more diffuse distribution around these key residues while preserving the overall protein architecture. The combination of increased conformational instability and direct active site interference correlates with the experimentally observed activity loss for DOSS complexes and suggests that SDS maintains a more compact, functional conformation under thermal stress.

These thermal stability findings further align with Fourier-transform infrared spectroscopy (FTIR) analysis reported in our previous work,^10^ which showed maintained α-helical content upon SDS complexation, consistent with the formation of a molten globule-like state with retained secondary structure but reduced tertiary organization.^33^ Molten globule states can demonstrate favorable non-native functionalities, including enhanced stability profiles and altered hydrophobicity, without complete loss of catalytic activity.^34,35^ The preservation of enzymatic function alongside enhanced thermal stability is a significant shift from the conventional stability-activity trade-off paradigm, where increased stability typically comes at the cost of reduced conformational flexibility and diminished catalytic efficiency. This suggests that rational surfactant complexation can access conformational states that optimize stability and function, a significant advantage for developing robust protein-based delivery systems that must withstand pharmaceutical processing conditions while maintaining therapeutic activity.

### Surface Modification Improves Lysozyme-Lipid Compatibility

Having established that SDS complexation maintains thermal stability while preserving enzymatic function, we next investigated how these surface modifications affect protein–lipid compatibility, a prerequisite for successful nanocarrier integration. To do so, we used hot-stage polarized microscopy to directly visualize lysozyme dissolution behavior in pharmaceutical lipids under processing-relevant conditions, offering real-time insights into protein–lipid interactions (Figure 2).^21^

**Figure 2:**
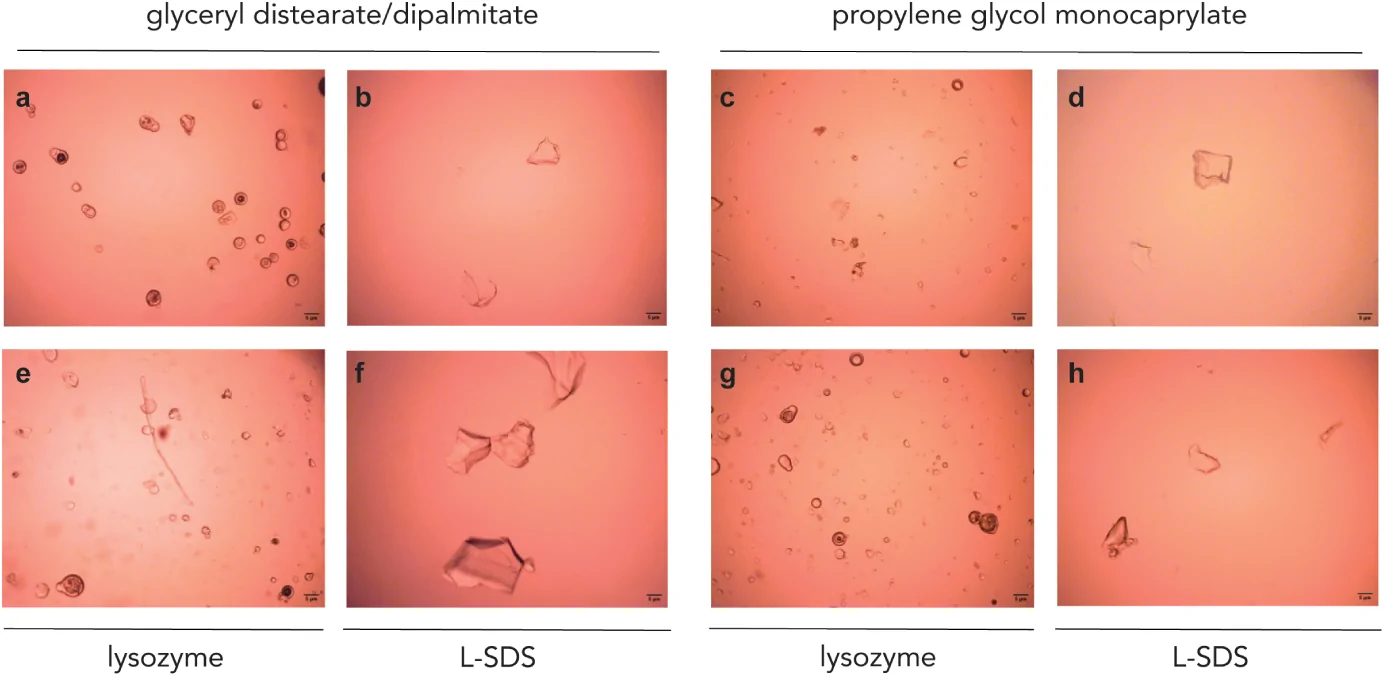
Lipid solubilization behavior of lysozyme and lysozyme-SDS complexes. Hot-stage polarized microscopy of native lysozyme and lysozyme-SDS complex (L-SDS) at 0.5 mg/mL (top row, **a–d**) and 1.0 mg/mL (bottom row, **e–h**) in glyceryl distearate/dipalmitate (solid lipid; **a**, **b**, **e**, **f**) and propylene glycol monocaprylate (liquid lipid; **c**, **d**, **g**, **h**). Birefringent structures indicate undissolved material. L-SDS complexes show reduced crystallization compared to native lysozyme at equivalent sample concentrations in both lipid phases, demonstrating enhanced lipid compatibility through surfactant complexation. Images were captured at 10× magnification.

The selected lipids, glyceryl dipalmitate/distearate (solid lipid) and propylene glycol monocaprylate (liquid lipid), are commonly used pharmaceutical excipients that serve complementary roles in nanostructured lipid carriers.^24,36,37^ The solid lipid provides structural matrix integrity, while the liquid lipid creates controlled imperfections that facilitate the drug accommodation and sustained release.

Native lysozyme formed characteristic crystalline structures in glyceryl dipalmitate/distearate even at concentrations as low as 0.5 mg/mL, indicating incomplete solubilization (Figure 2a,c). This limited saturation solubility reflects the thermodynamically unfavorable interactions between lysozyme’s predominantly hydrophilic surface and the lipid environment. The persistence of ordered structures under polarized light confirmed that native lysozyme cannot effectively integrate into lipid matrices without surface modification.

By comparison, SDS complexation showed a more pronounced transformation in solubility behavior (Figure 2b,d,f,h). Lysozyme-SDS complexes showed reduced crystallization tendency, forming less numerous and less birefringent structures compared to native lysozyme at equivalent concentrations. Quantitatively, complexes dissolved at concentrations up to 1.0 mg/mL in glyceryl dipalmitate/distearate (containing 0.73 mg/mL lysozyme based on complex stoichiometry), representing a 50% improvement over native lysozyme’s saturation solubility (0.5 mg/mL). Similar enhancements were observed in propylene glycol monocaprylate, indicating broad improvements in lipid miscibility across both solid and liquid lipid components (Figure 2; Supporting Information Figures S5 and S6).

The enhanced lipid solubility of lysozyme-SDS complexes reflects fundamental changes in protein surface properties that overcome natural protein–lipid incompatibilities. Hydrophobic ion pairing creates amphiphilic domains where SDS alkyl chains extend outward from lysozyme’s surface, presenting a hydrophobic interface to the lipid environment. Simultaneously, charge neutralization through ionic interactions between SDS sulfate groups and lysozyme’s cationic residues reduces electrostatic repulsion at the protein–lipid interface.

This improved protein solubility provides substantial benefits for high-concentration formulations, reduces the lipid carrier volume required for therapeutic doses, and directly addresses the central challenge of integrating hydrophilic proteins into lipophilic materials. These compatibility improvements were pivotal for superior nanocarrier integration, which we investigated next through systematic NLC formulation studies.

### Superior NLC Integration through Matrix Engineering

To ensure formulation stability and maximize protein loading, native lysozyme and lysozyme-SDS complexes were incorporated at their respective saturation solubility limits (0.5 mg/mL and 1.0 mg/mL), resulting in different total protein inputs (61.5 μg for native lysozyme, 123 μg for lysozyme-SDS).

Under these optimized formulation conditions, lysozyme-SDS complexes showed improved encapsulation efficiency compared to native lysozyme (79.6 ± 1.8% vs 18.4 ± 4.3%, *p* < 0.001, Welch’s *t*-test), representing a 4-fold improvement. Drug loading similarly improved from 8.8 ± 2.0 μg/mg to 55.5 ± 1.3 μg/mg (*p* < 0.0001, Welch’s *t*-test), a 6-fold enhancement. Notably, lysozyme-SDS formulations also showed improved batch-to-batch reproducibility (coefficient of variation: 2.3% vs 23.3% for encapsulation efficiency), suggesting more consistent and predictable protein–lipid integration, a critical advantage for pharmaceutical manufacturing.

Nanoparticle size and surface charge properties provide complementary insight into localization patterns within the nanocarrier structure (Figure 3). These physicochemical properties reflect how proteins interact with the lipid matrix architecture and can distinguish between surface-associated and matrix-incorporated protein.

**Figure 3:**
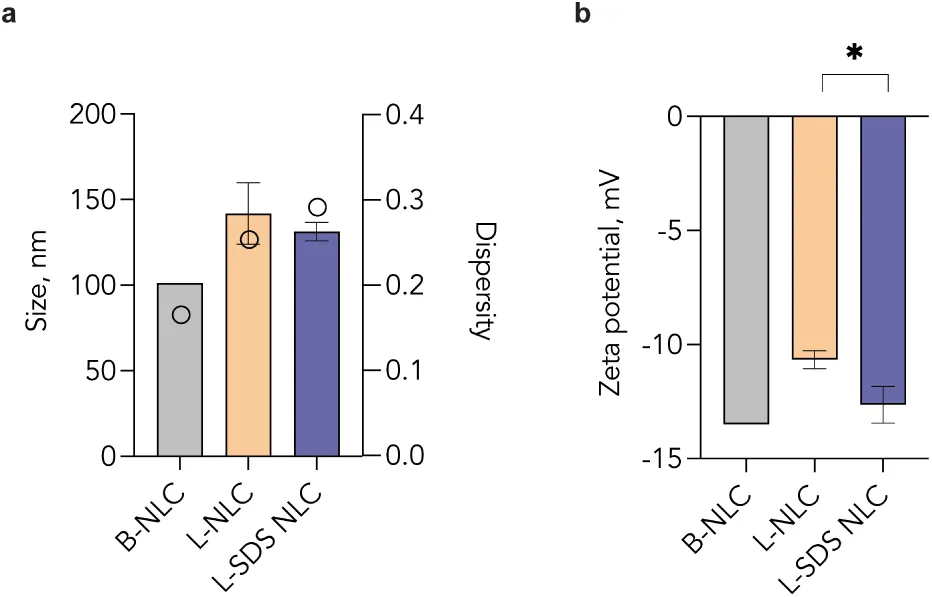
Surfactant complexation leads to altered NLC physicochemical properties. **a** Particle size (bars, left *y*-axis) and polydispersity index (open circles, right *y*-axis) of blank NLCs (B-NLC), lysozyme-loaded NLCs (L-NLC), and lysozyme-SDS complex-loaded NLCs (L-SDS NLC). **b** Zeta potential measurements of the three formulations. Data presented as mean ± SD (*n* = 3, independent triplicates). Statistical comparisons between L-NLC and L-SDS NLC were performed using Welch’s *t*-test on batch means. *, *p* < 0.05.

Blank NLCs showed a mean hydrodynamic diameter of 101.3 ± 1.5 nm with low dispersity and a negative surface charge (−13.5 ± 0.7 mV), consistent with polysorbate 80 stabilization (Figure 3a,b). Incorporation of native lysozyme resulted in an apparent increase in particle size (141.9 ± 18.0 nm), whereas lysozyme-SDS complex incorporation showed a more moderate increase (128.1 ± 2.4 nm). However, this particle size difference between protein-loaded NLCs did not reach statistical significance (Welch’s *t*-test, *p* = 0.31). Equally, dispersity values were comparable between formulations (*p* = 0.27), indicating that protein incorporation did not measurably affect dispersity.

Zeta potential measurements provided further insight into protein localization. Blank NLCs carried the most negative surface charge (−13.5 ± 0.7 mV), with L-SDS NLCs showing a comparable value (−12.6 ± 0.8 mV), while L-NLCs were the least negative (−10.9 ± 0.8 mV; *p* = 0.034 vs L-SDS NLC). The similarity between blank and L-SDS NLCs is inconsistent with significant surface localization of the negatively charged complex and instead suggests predominantly internal accommodation within the lipid matrix, while the less negative zeta potential of L-NLCs aligns with surface association of the cationic native protein (pI ∼ 11). These findings are interpreted alongside the DSC crystallinity data below.

### Thermal Analysis Shows Molecular Basis of Enhanced Nanocarrier Integration

To understand the molecular mechanisms underlying the observed encapsulation improvements, we next used differential scanning calorimetry (DSC) for a detailed thermal characterization. These analyses provided critical insights into protein conformational states and their effect on NLC encapsulation.

### Protein Conformational Analysis

DSC thermograms of native and complexed lysozyme showed distinct thermal signatures that correlate with their functional properties (Figure 4a; Supporting Information Tables S1 and S2). Native lysozyme showed a denaturation profile with a melting temperature (*T*_m_) of 113.4 ± 4.0 °C and a denaturation enthalpy (Δ*H*) of 38.2 ± 6.8 J/g, consistent with its compact, well-folded tertiary structure and the robust thermal stability observed in activity assays at 70 °C.^38^

**Figure 4:**
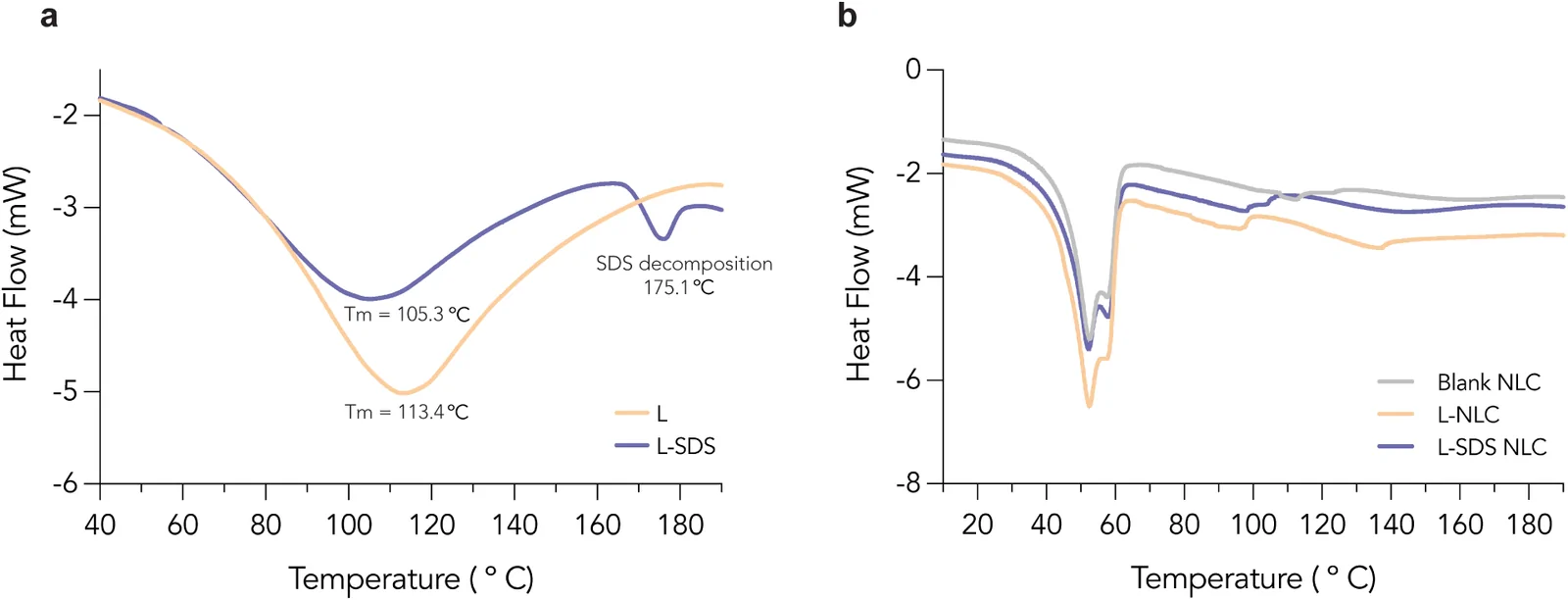
Thermal analysis demonstrates enhanced stability and reduced crystallinity mechanisms. **a** DSC thermogram comparing native lysozyme (L, yellow) with lysozyme-SDS complex (L-SDS, purple) showing enhanced thermal stability of the lysozyme-SDS complex with the SDS decomposition peak at 175.1 °C confirming stable binding. **b** DSC thermograms showing melt transitions for blank NLC (gray), lysozyme-loaded NLC (yellow), and lysozyme-SDS-loaded NLC (purple). The reduction in peak intensity correlates with decreased crystallinity upon protein incorporation. Endotherms are plotted downward; enthalpies are reported with the instrument sign convention.

Lysozyme-SDS complexation resulted in notable changes in the thermal profile. The *T*_m_ decreased to 105.3 ± 4.8 °C, while the denaturation enthalpy decreased to 21.8 ± 2.7 J/g, indicative of a conformational change, consistent with our previous findings that align with the formation of a molten globule-like state.^10^ An additional thermal event at 175.1 ± 0.5 °C, distinct from both native lysozyme denaturation and the expected melting point of pure SDS (204–207 °C). This intermediate thermal transition is consistent with the formation of a lysozyme-SDS complex, as protein–surfactant interactions are known to modify thermal transitions and create distinct DSC peaks.^39,40^

The conformational flexibility implied by this molten-globule-like state likely facilitates protein integration into lipid matrices by enabling adaptive surface interactions and reducing steric constraints during nanocarrier formation. Importantly, the protein maintains biological activity while adopting a more thermodynamically compatible structure for the lipid incorporation.

### Protein Integration within NLC Matrix

DSC analysis of lyophilized NLC formulations showed that protein incorporation was successful within the lipid organization, with the extent of its solubility compatibility correlating with encapsulation performance (Figure 4b, Supporting Information, Tables S1 and S2).

Blank NLCs showed characteristic melting profiles with well-defined endothermic transitions at 52.3 ± 0.0 °C and a melting enthalpy of 12.3 ± 1.7 J/g, corresponding to 63.0% relative crystallinity (calculated using pure glyceryl distearate/dipalmitate, Δ*H*_m_ = 28.3 ± 1.0 J/g, as a reference). This reduction from 100% reflects the expected effects of liquid lipid incorporation and nanostructuring.

Native lysozyme incorporation slightly increased lipid crystallinity to 68.6%, suggesting limited integration within the lipid matrix or surface localization. In contrast, lysozyme-SDS complexes reduced crystallinity to 56.8%, indicating matrix disruption, and improved protein incorporation. This is consistent with lysozyme-SDS formulations showing superior encapsulation efficiency (79.6% vs 18.4%), suggesting that amphiphilic surface properties enable more effective protein accommodation within the lipid matrix while maintaining sufficient structural integrity for controlled release applications. Moreover, the lack of an SDS decomposition peak observed with the L-SDS NLC peak at 175.1 ± 0.5 °C, and the similarity of the heat flow peak compared to the blank NLC DSC thermogram suggests successful integration within the lipid matrix (Figure 4b).

To evaluate the functional consequences of this enhanced matrix integration, we next assessed the protein release kinetics and enzymatic activity retention under simulated gastrointestinal conditions.

### Lysozyme Release and Stability in Simulated Gastrointestinal Fluids

Protein release studies were performed in an enzyme-free simulated gastric fluid (SGF, pH 1.2) and simulated intestinal fluid (SIF, pH 6.8) to assess the effect of pH on protein release and stability independent of enzymatic degradation. Enzymatic activity was measured at each time point to determine whether the released lysozyme retained its biological function.

### Release Behavior in Simulated Gastric Conditions

In SGF, both formulations showed rapid initial release within 30 min (36.6 ± 12.6% and 39.5 ± 9.2% for L-NLC and L-SDS NLC, respectively, Figure 5a). This burst release likely reflects surface-associated protein or the limited stability of the lipid matrix under acidic conditions. Lysozyme-SDS NLC maintained relatively stable release throughout the study period (39.9 ± 2.9% at 2 h), whereas lysozyme-loaded NLC showed a modest reduction in detectable protein over time (31.8 ± 14.4% at 2 h).

**Figure 5:**
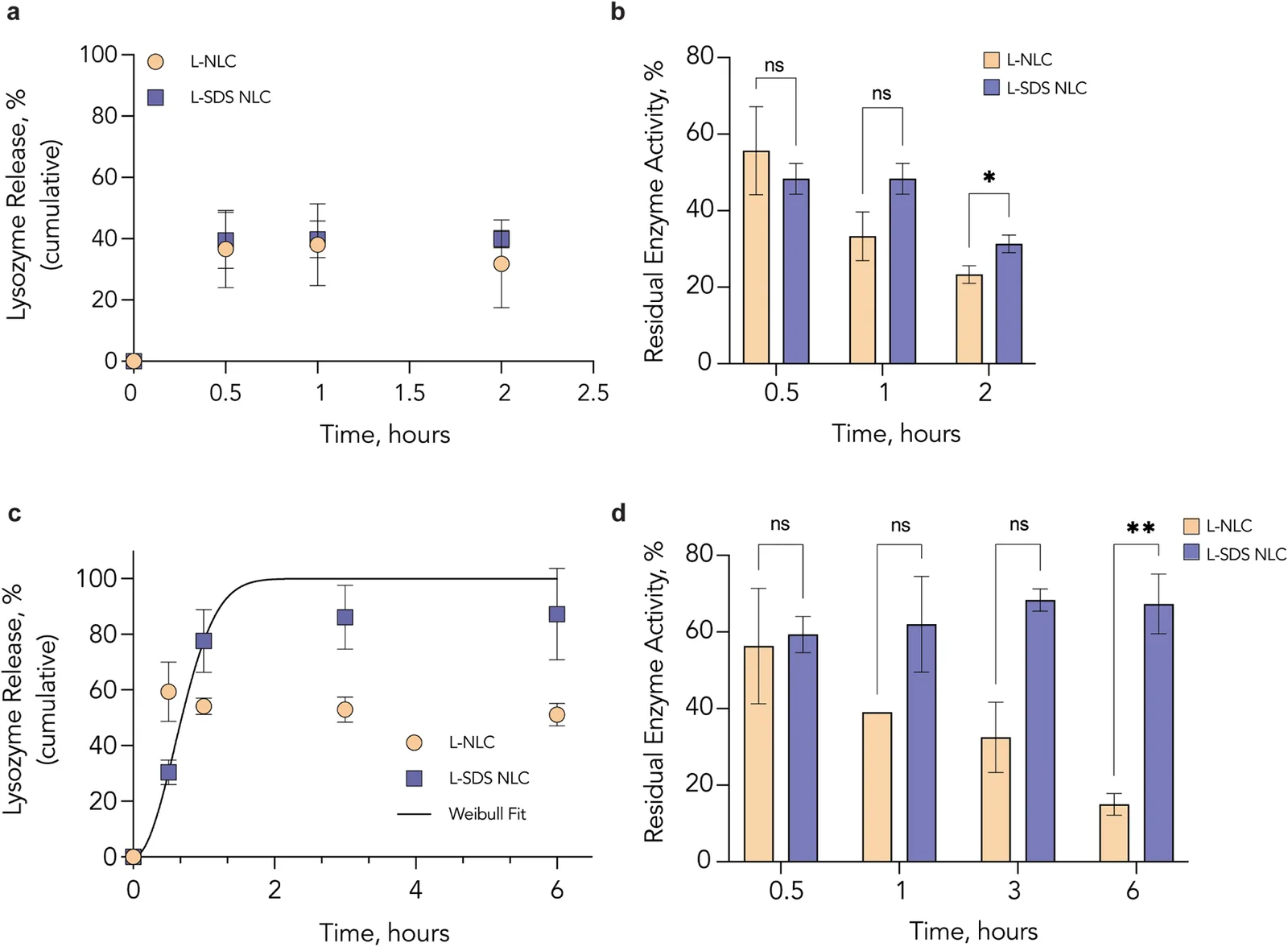
Release profiles and retained enzymatic activity of lysozyme from NLC formulations in simulated gastrointestinal fluids. **a** Cumulative lysozyme release in SGF, pH 1.2. **b** Residual enzymatic activity in SGF, expressed as a percentage relative to native lysozyme at equivalent concentration. **c** Cumulative lysozyme release in SIF, pH 6.8. For clarity, a Weibull fit is shown only for L-SDS release in SIF, which showed sufficient time-dependent curvature to support kinetic modeling. **d** Residual enzymatic activity in SIF, expressed as a percentage relative to native lysozyme at equivalent concentration. Data represent mean ± SD (*n* ≥ 2 independent batches). Statistical analysis was performed using two-way ANOVA with Šídák’s multiple comparisons test. *, *p* < 0.05, **, *p* < 0.01, ns = not significant.

The decline in detectable lysozyme from L-NLC formulations corresponded with a progressive loss of enzymatic activity (Figure 5b). Two-way repeated measures ANOVA confirmed a significant effect of time (*p* = 0.0018), consistent with ongoing degradation in SGF. Both formulations showed comparable initial activity retention, though lysozyme-SDS NLCs trended toward improved stability at the 2-h time point (*p* = 0.0469, Šídák-adjusted). This observation is consistent with reports that hydrophobic ion pairs remain stable in simulated gastric environments due to maintained electrostatic interactions between ionized protein residues and surfactant headgroups at low pH.^8,9^ In contrast, native lysozyme activity decreased to approximately 20% by 2 h, suggesting aggregation or degradation in the acidic, high ionic strength environment. While lysozyme can resist acid-induced unfolding at pH 1.2,^41^ the high ionic strength of SGF may promote protein aggregation.

### Release Behavior in Simulated Intestinal Conditions

In SIF, distinct release behaviors were observed (Figure 5c). L-SDS NLC demonstrated time-dependent release, reaching 30.4 ± 4.5% at 30 min, plateauing at approximately 3 h, with a final release of 87.2 ± 16.5%. In contrast, L-NLC showed an initial burst release (59.4 ± 10.6%) followed by a decline in detectable lysozyme over 6 h (51.1 ± 4.1%).

Under intestinal conditions, the L-SDS formulation showed sustained release behavior well-described by Weibull kinetics (β = 2.04, *R* ^2^ = 0.89), with the shape parameter indicating sigmoidal/complex release consistent with time-dependent matrix effects. First-order analysis corroborated this mechanism, yielding a release half-time of 0.66 h. In contrast, L-NLC release kinetics could not be adequately modeled, consistent with the observed burst release and subsequent protein degradation (Supporting Information, Tables S6 and S7).

The protective effect of hydrophobic ion pairing was most pronounced under intestinal conditions (Figure 5d). Initial activity at 30 min was comparable between formulations (L-NLC: 56.5 ± 15.1%, L-SDS NLC: 59.4 ± 4.6%; Šídák-adjusted *p* > 0.05), but the activity profiles diverged progressively over time. Mixed-effects analysis showed a highly significant main effect of formulation (*p* < 0.0001) and a significant time-dependent interaction (*p* = 0.0075), indicating that the magnitude of protection increased during intestinal exposure. By the final time point, L-SDS retained 67.5 ± 7.5% activity, whereas L-NLC retained only 15.2 ± 3.0% (Šídák-adjusted *p* = 0.0097). This pronounced late-stage separation highlights the stabilizing influence of SDS complexation in intestinal pH and supports improved structural preservation of the protein within the lipid matrix.

These findings suggest that HIP enhances the retention of lysozyme within the lipid matrix and promotes conformational stability upon release, resulting in the delivery of functionally active protein under simulated gastrointestinal conditions.

## Conclusions

This study demonstrates that hydrophobic ion pairing enables the rational engineering of protein–lipid interfaces for enhanced nanocarrier integration. Controlled lysozyme-SDS complexation simultaneously improved thermal stability at processing-relevant temperatures, lipid compatibility, and encapsulation efficiency, while preserving enzymatic function through simulated gastrointestinal transit.

Structural characterization demonstrated that surfactant complexation leads to the formation of a molten globule-like state that preserves bioactivity while imparting amphiphilicity for superior material compatibility. Strategic reduction of lipid matrix crystallinity shows how lysozyme-SDS complexes create optimal accommodation spaces within carrier systems, providing insights into the molecular determinants of protein–lipid compatibility.

Our findings establish broadly applicable design principles for protein delivery systems, where controlled conformational flexibility enhances both stability and activity, while amphiphilic surface engineering overcomes natural protein–lipid incompatibilities. The preservation of enzymatic function throughout modification, thermal processing, and simulated gastrointestinal exposure addresses critical challenges in oral protein delivery. While this study focuses on lysozyme as a model protein, the principles demonstrated, including enhanced lipid solubility through charge neutralization, improved matrix integration via crystallinity disruption, and functional preservation through complexation, may extend to other cationic proteins with similar structural features. Future work will assess the generality of this approach across structurally diverse therapeutic proteins under enzymatic gastrointestinal conditions.

## Supplementary Material



## Data Availability

Data for this article are available at the Queen’s University Research Portal.

## Notes

Standard curve for lysozyme quantification by cation exchange chromatography; molecular dynamics simulation data including secondary structure histograms, RMSD profiles, and radial distribution functions for lysozyme, lysozyme-SDS, and lysozyme-DOSS systems at 70 °C; hot-stage polarized light microscopy images of lysozyme and lysozyme-SDS complex in glyceryl distearate/dipalmitate and propylene glycol monocaprylate; DSC melting point, melting enthalpy, crystallization point, and crystallization enthalpy data for all formulations and individual components; upscaled NLC formulation compositions for encapsulation efficiency and release studies; Weibull and first-order kinetic model parameters for cumulative lysozyme release in SGF and SIF; hydrodynamic particle size of lysozyme and lysozyme-SDS complex in 1.5% w/v polysorbate 80 for ultrafiltration membrane selection (PDF)
