# Supplementary material for: Hydrophobic Ion Pairing Improves Protein Thermal Stability and Lipid Nanocarrier Integration through Controlled Surface Modification
Source: ACS Nanosci Au. 2026 May 12;6(4):644–53. doi: 10.1021/acsnanoscienceau.6c00033 (PMC13495457; doi:10.1021/acsnanoscienceau.6c00033)
Supplement: Supplementary file 1 [file ng6c00033_si_001.pdf]

Supporting Information

Hydrophobic Ion Pairing Improves Protein Thermal Stability and Lipid Nanocarrier Integration Through Controlled Surface Modification

Jiaming Mu[1], Daniel Sedough-Abbasian[1], Leran Mao[2], and Sheiliza Carmali [1,*]

[1]School of Pharmacy, Queen's University Belfast, BT9 7BL, United Kingdom

[2] Department of Chemical Engineering, Carnegie Mellon University, Pittsburgh, Pennsylvania, 15213, United States

* Corresponding author:

E-mail: s.carmali@qub.ac.uk (SC)

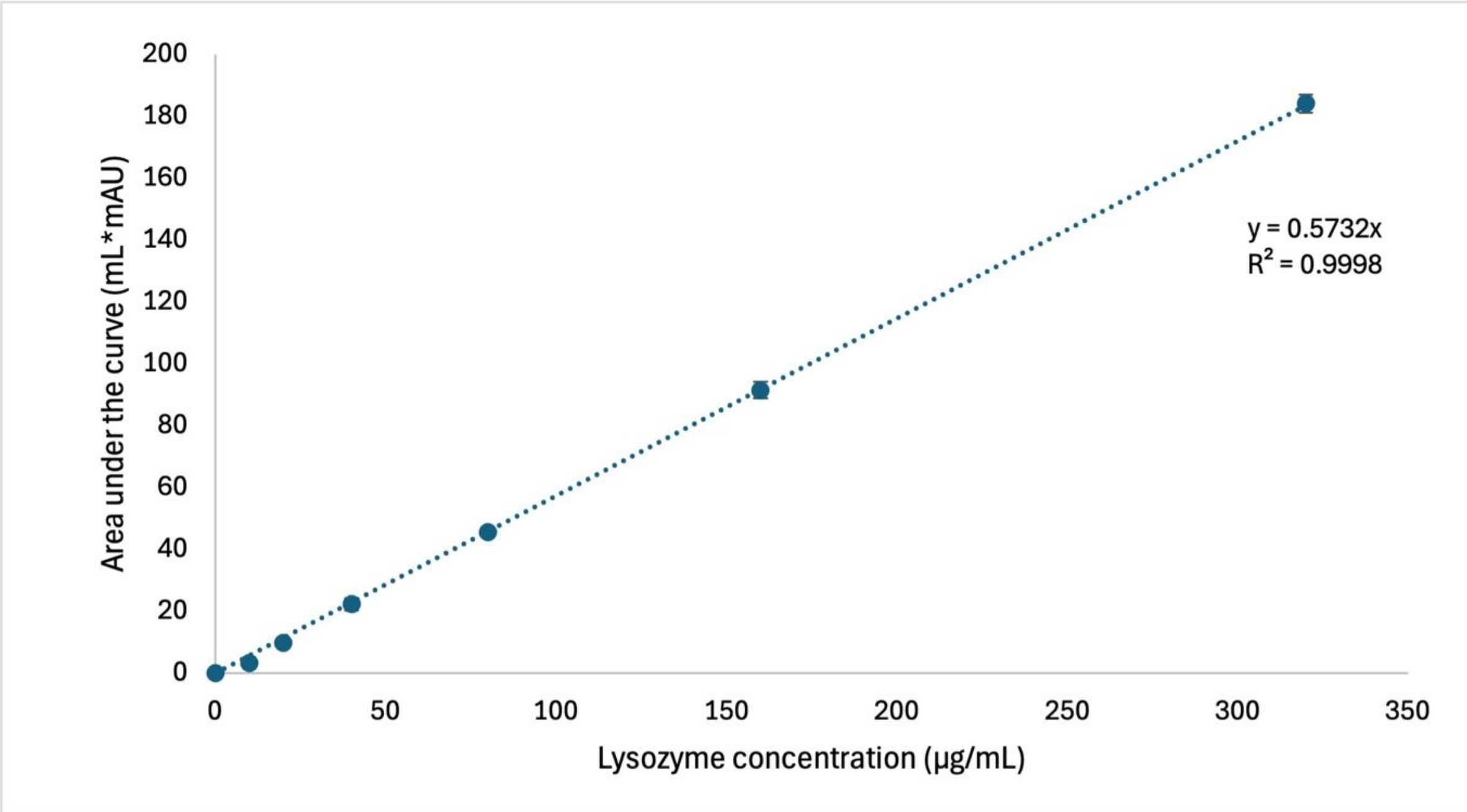

Figure S1 Standard curve for lysozyme used for quantification of encapsulation efficiency and drug loading.

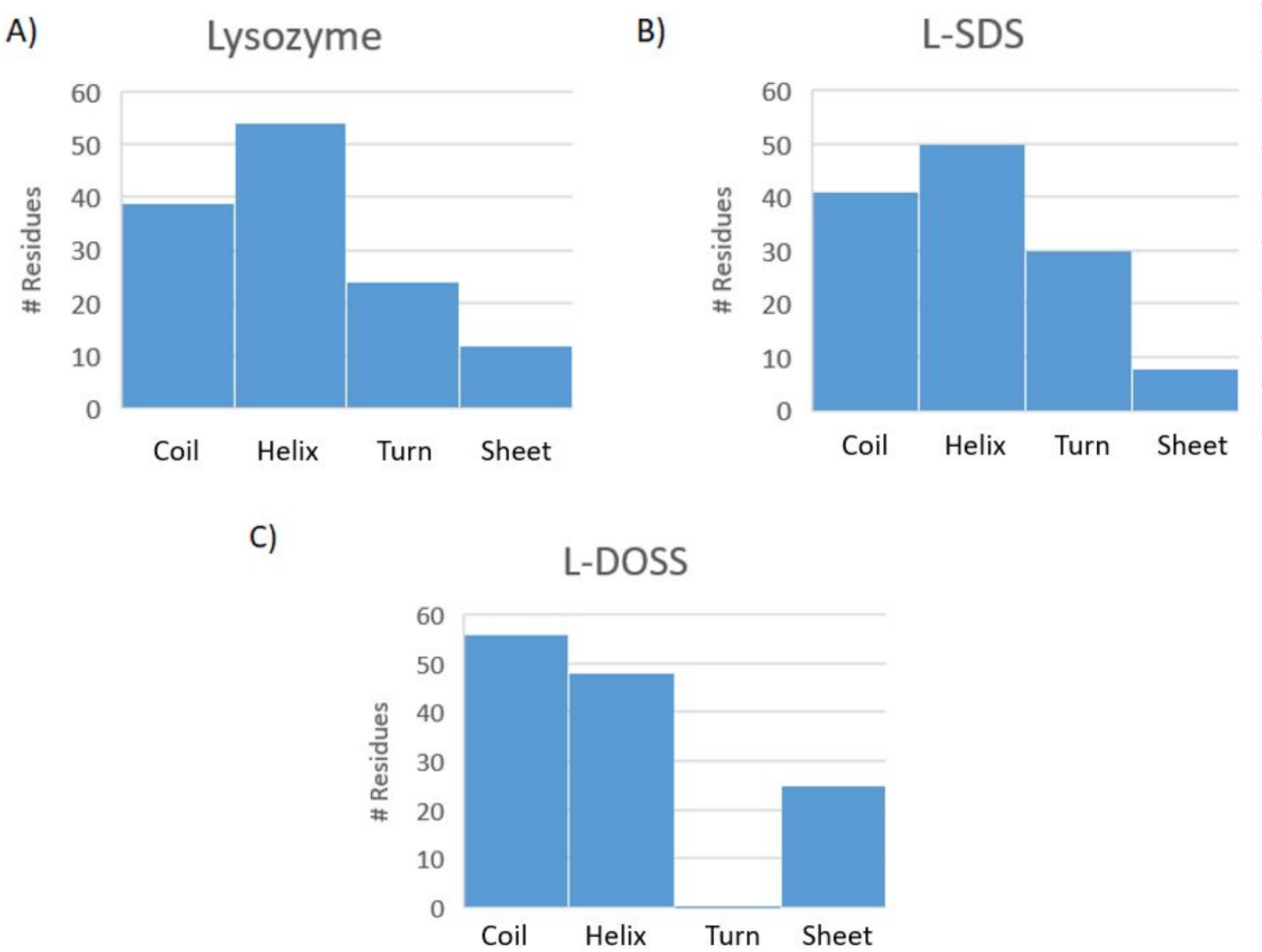

Figure S2 Histogram of secondary structure in the final 5 ns frame in MD simulations of A) lysozyme, B) lysozyme with 18 SDS molecules and C) lysozyme with 18 DOSS molecules.

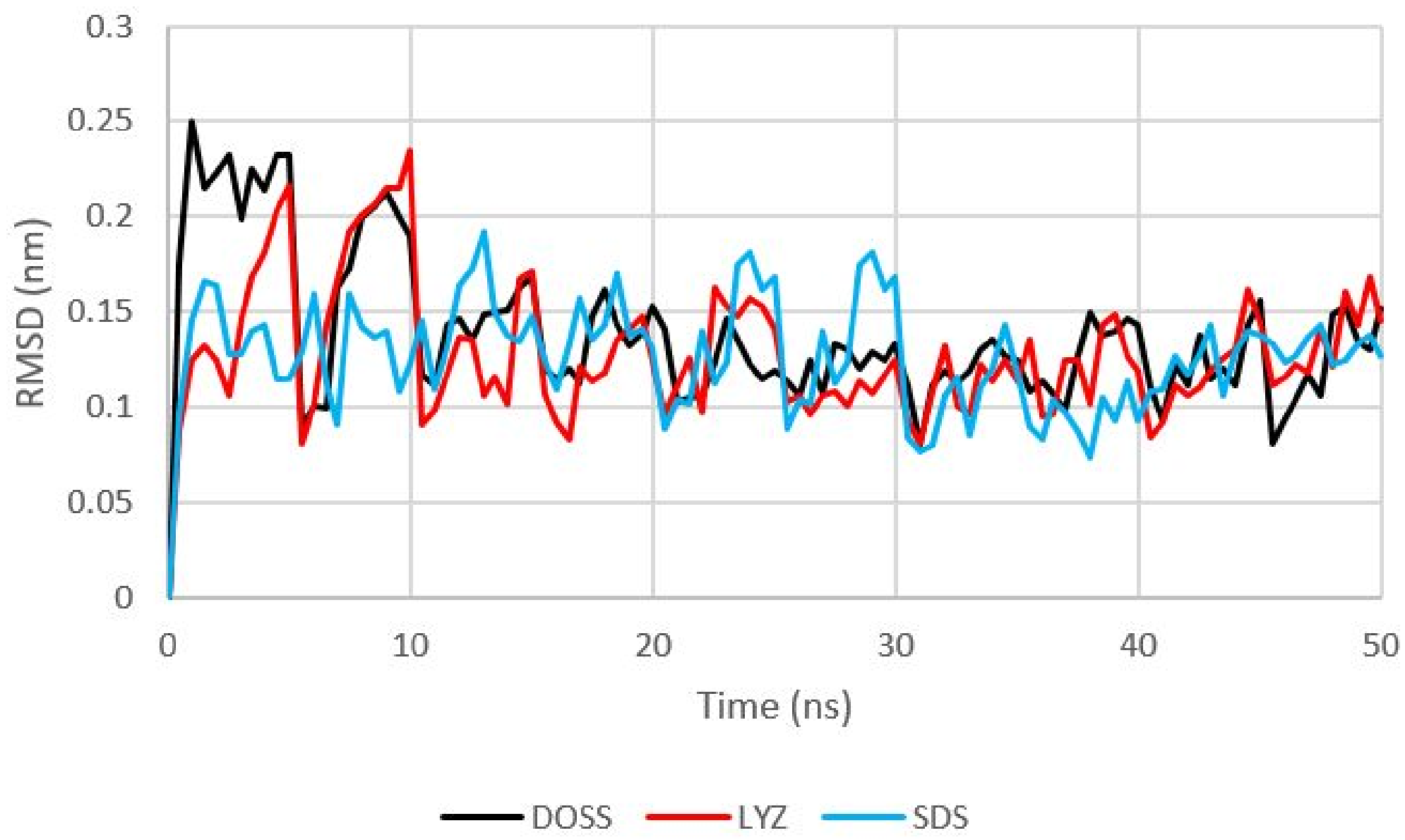

Figure S3 Root-mean-squared deviation (RMSD) of tested experimental conditions. The black, red, blue, and green lines represent lysozyme complexed with DOSS, native lysozyme, and lysozyme complexed SDS molecules. 18 molecules of SDS and DOSS were added to the simulation at 70 ^{o}C.

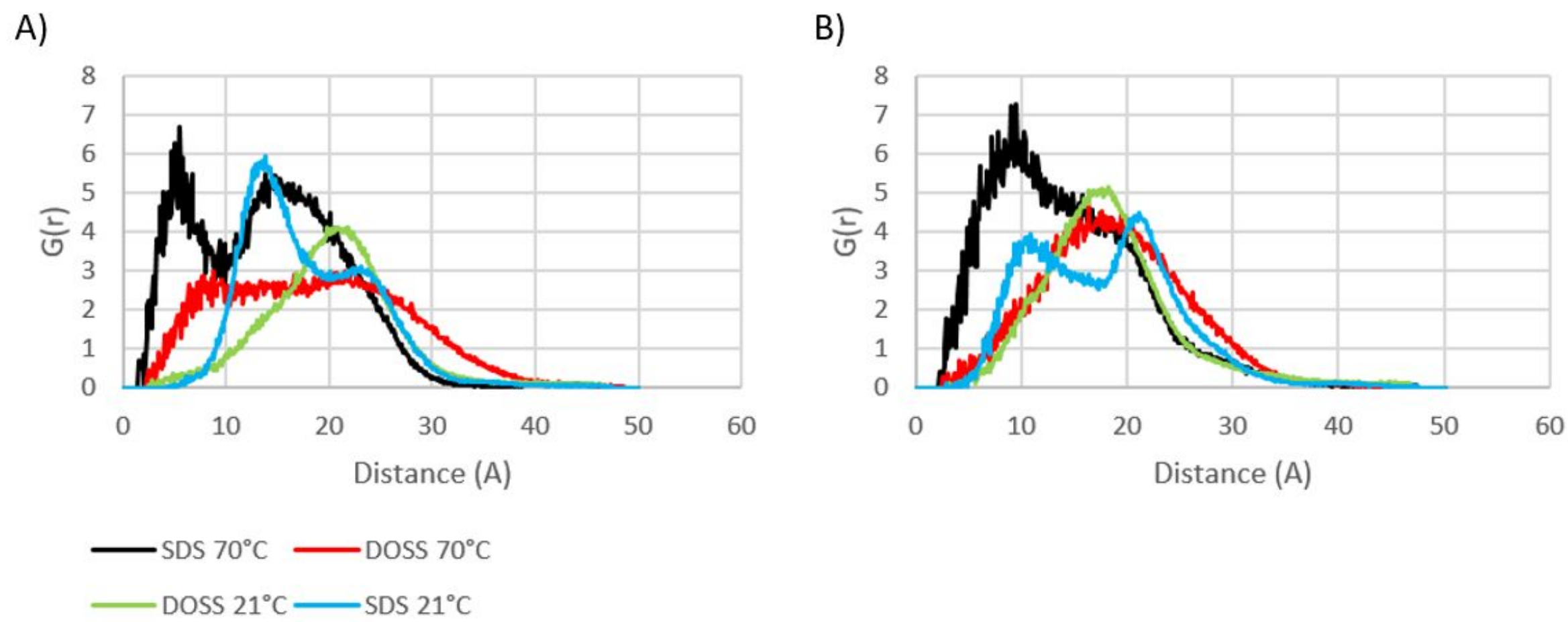

Figure S4. Radial distribution function of surfactant molecules around the active catalytic sites A) Glu35 and B) Asp52 at different temperatures.

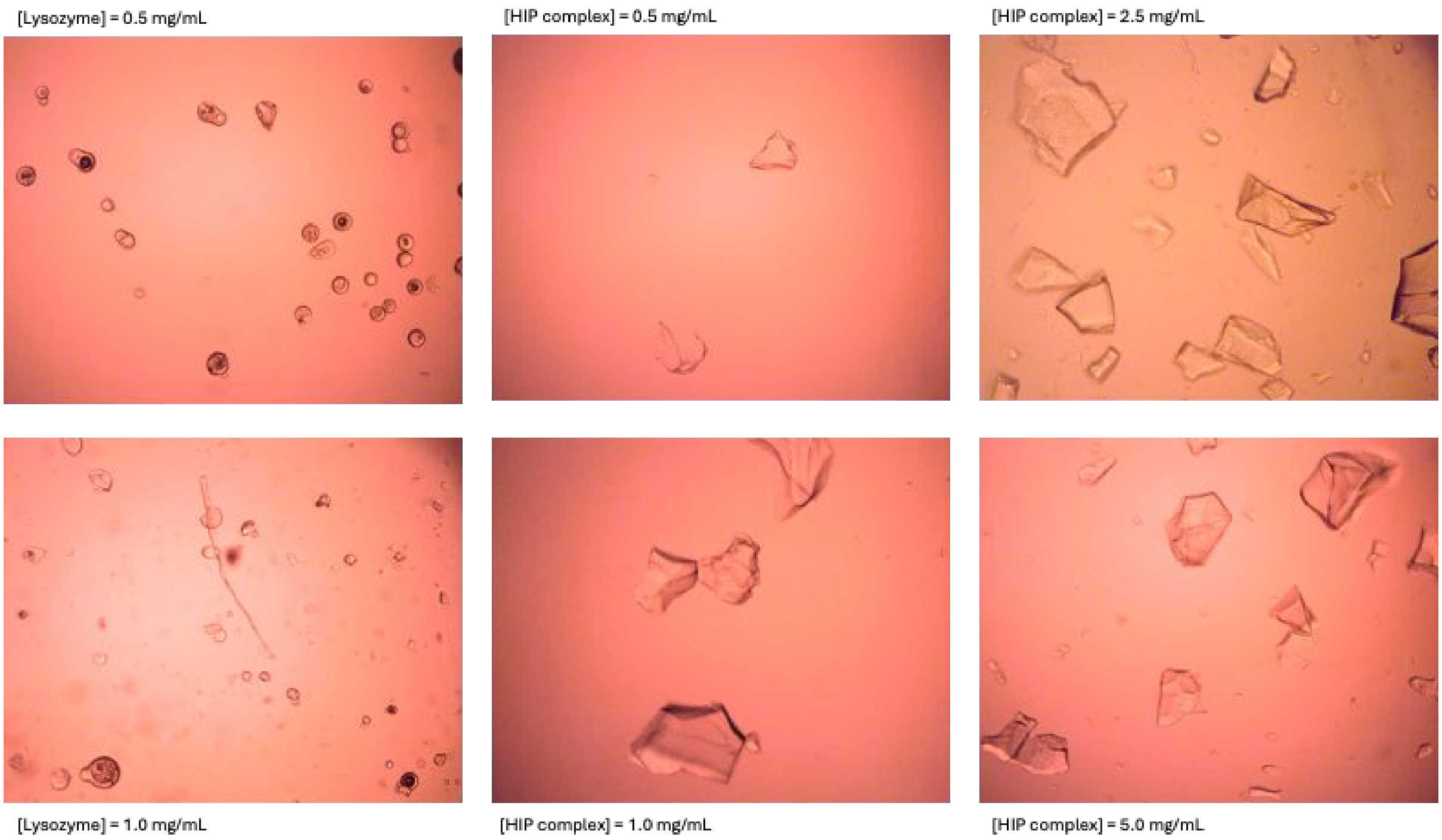

Figure S5. Lipid solubilisation behaviour of lysozyme and lysozyme-SDS (HIP complex) in glyceryl distearate/dipalmitate.

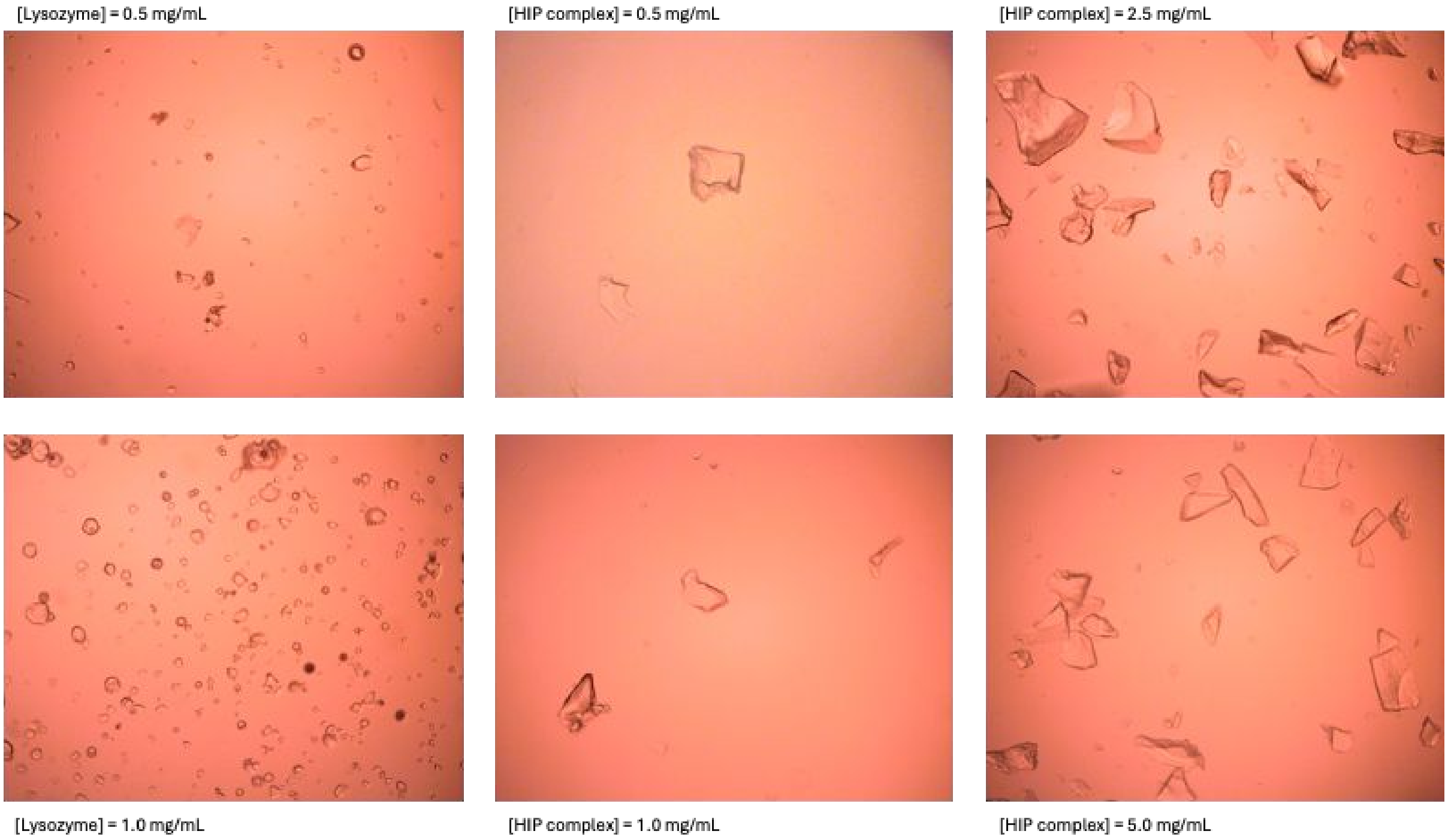

Figure S6. Lipid solubilisation behaviour of lysozyme and lysozyme-SDS (HIP complex) in propylene glycol monocaprylate.

Table S1 Melting point data obtained from DSC. Represented as mean ± standard deviation

	Peak 1	Peak 2	Peak 3
Native Lyz	113.4 ± 4.0		
HIP complex	105.3 ± 4.8		175.1 ± 0.5
Solid lipid	60.3 ± 0.5		
Physical lipid mixture	50.0 ± 4.7		155.0 ± 10.5
Blank NLC	52.3 ± 0.0	112.9 ± 9.8	149.5 ± 9.9
Lyz NLC	52.6 ± 0.4	97.8 ± 10.7	138.4 ± 3.1
HIP NLC	52.0 ± 0.4	104.5±7.6	149.7 ± 8.1

Table S2 Melting enthalpy data obtained from DSC. Represented as mean ± standard deviation

	Peak 1	Peak 2	Peak 3
Native Lyz	38.2 ± 6.8		
HIP complex	21.8 ± 2.7		0.8 ± 0.1
Solid lipid	28.3 ± 1.0		
Physical lipid mixture	16.3 ± 1.8		12.6 ± 2.5
Blank NLC	12.3 ± 1.7	2.4 ± 1.6	1.4 ± 0.2
Lyz NLC	13.4 ± 1.1	4.1 ± 0.4	2.3 ± 0.7
HIP NLC	11.1 ± 0.7	2.5 ± 1.0	2.1 ± 0.5

Table S3 Crystallisation point data obtained from DSC.

	Rep 1	Rep 2	Rep 3	Mean ± STD
Solid lipid	51.2	50.4	50.8	50.8 ± 0.4
Physical lipid mixture	50.8	50.5	50.8	50.7 ± 0.2
Blank NLC	46.4	47.0	46.7	46.7 ± 0.3
Lyz NLC	46.7	46.9	46.7	46.7 ± 0.1
HIP NLC	47.2	46.7	46.2	46.7 ± 0.5

Table S4 Crystallisation enthalpy data obtained from DSC

	Rep 1	Rep 2	Rep 3	Mean ± STD
Solid lipid	18.6	17.6	18.1	18.1 ± 0.5
Physical lipid mixture	13.0	12.0	12.5	12.5 ± 0.5
Blank NLC	6.9	7.7	8.1	7.6 ± 0.6
Lyz NLC	8.6	8.3	7.4	8.1 ± 0.6
HIP NLC	6.3	6.6	7.1	6.7 ± 0.4

Table S5 Component of upscaled nanostructured lipid carriers for EE% and release study

Component	Blank NLCs	L-NLCs	L-SDS NLCs
Lipid phase			
Glyceryl distearate/dipalmitate	722.5 μL	722.5 μL	722.5 μL
Propylene glycol monocaprylate	323 μL	323 μL	323 μL
Lysozyme	-	0.5 mg	-
Lysozyme-SDS complex	-	-	1 mg
Aqueous phase			
Polysorbate 80	510 mg	510 mg	510 mg
Water	34 mL	34 mL	34 mL

Table S6 Weibull model parameters obtained by nonlinear regression of cumulative lysozyme release profiles in simulated gastric fluid (SGF) and simulated intestinal fluid (SIF). Values of *b* < 1 are associated with burst-dominated or decelerating release, *b* ≈ 1 corresponds to approximately exponential release kinetics, and *b* > 1 indicates sigmoidal or complex, non-Fickian release behaviour. Parameters were constrained to positive values. In cases where release occurred predominantly at early time points followed by an extended plateau, the scale parameter *a* was not uniquely identifiable and is reported as unstable, reflecting limited time-dependent curvature rather than poor model convergence. Mechanistic interpretation was therefore restricted to datasets exhibiting identifiable parameters and adequate goodness of fit.

	Medium	a (h)	b	95% CI (a)	95% CI (b)	R^2	Notes
L-NLC	SGF	5.36×10^{108}	0.0033	-	-	-	Burst-dominated release
L-SDS NLC	SGF	Unstable	0.0125	Very wide	~0–0.229	0.932	Early burst; parameters not identifiable
L-NLC	SIF	Unstable	0.0007	Very wide	0.00034–0.071	0.953	Rapid initial release
L-SDS NLC	SIF	0.821	2.04	0.677–1.340	0.528–3.435	0.888	Time-dependent release

Table S7 First-order kinetic parameters obtained by fitting cumulative lysozyme release profiles to a one-phase association model, where *k* is the apparent first-order release rate constant. The corresponding half-time ($t_{1/2}$) represents the time required to reach 50% of the asymptotic release and is calculated as ln(2)/k. Fits were performed with the initial release fixed at zero and the plateau constrained to 100% cumulative release. Goodness of fit is reported as R^2. Low or negative R^2 values indicate that release is not adequately described by a simple first-order process and typically reflect release dominated by an initial burst rather than gradual, time-dependent release.

	Medium	k (h^{-1})	$t_{1/2}$ (h)	R^2	Notes
L-NLC	SGF	0.324	2.14	0.10	Poor fit; burst release
L-SDS NLC		0.397	1.75	0.35	Poor–moderate fit
L-NLC	SIF	0.305	2.28	−0.62	Model inappropriate
L-SDS NLC		1.05	0.66	0.86	Good first-order behaviour

Table S8 Hydrodynamic particle size of lysozyme and lysozyme–SDS complex in 1.5% w/v polysorbate 80 solution, as determined by dynamic light scattering. Particle size characterisation was performed to inform ultrafiltration membrane selection for encapsulation efficiency determination, confirming that neither species exceeds the ~20 nm pore size of the 1000 kDa MWCO membrane (Vivaspin® 20) used for separation of non-encapsulated protein from intact NLCs.

	**Lysozyme in 1.5% w/v polysorbate 80**	**L–SDS complex in 1.5% w/v polysorbate 80**
**Particle size (nm)**	9.2 ± 0.1	13.3 ± 0.7
